# NiFe_2_O_4_/Ketjen Black Composites as Efficient Membrane Separators to Suppress the Shuttle Effect for Long-Life Lithium-Sulfur Batteries

**DOI:** 10.3390/nano12081347

**Published:** 2022-04-14

**Authors:** Wen Jiang, Lingling Dong, Shuanghui Liu, Shuangshuang Zhao, Kairu Han, Weimin Zhang, Kefeng Pan, Lipeng Zhang

**Affiliations:** 1 School of Chemistry and Chemical Engineering, Shandong University of Technology, Zibo 255049, China; wengejang@126.com (W.J.); donglingling202107@163.com (L.D.); shuanghuiliu2020@163.com (S.L.); hankairu2022@126.com (K.H.); wmzhang@sdut.edu.cn (W.Z.); 2 School of Materials and New Energy, South China Normal University, Shanwei 516600, China; 20219207@m.scnu.edu.cn

**Keywords:** lithium-sulfur battery, nano NiFe_2_O_4_, adsorption, separator modification

## Abstract

Lithium-sulfur batteries exhibit great potential as one of the most promising energy storage devices due to their high theoretical energy density and specific capacity. However, the shuttle effect of the soluble polysulfide intermediates could lead to a severe self-discharge effect that hinders the development of lithium-sulfur batteries. In this paper, a battery separator has been prepared based on NiFe_2_O_4_/Ketjen Black (KB) modification by a simple method to solve the shuttle effect and improve the battery performance. The as-modified separator with the combination of small-size KB and NiFe_2_O_4_ nanoparticles can effectively use the physical and chemical double-layer adsorption to prevent polysulfide from the shuttle. Moreover, it can give full play to its catalytic effect to improve the conversion efficiency of polysulfide and activate the dead sulfur. The results show that the NiFe_2_O_4_/KB-modified separator battery still maintains a discharge capacity of 406.27 mAh/g after 1000 stable cycles at a high current density of 1 C. Furthermore, the coulombic efficiency remains at 99%, and the average capacity attenuation per cycle is only 0.051%. This simple and effective method can significantly improve the application capacity of lithium-sulfur batteries.

## 1. Introduction

The demand for a diverse and comprehensive transformation of the energy structure based on fossil energy for clean and renewable energy is becoming stronger and stronger in the energy field [1]. The electrochemical energy storage strategy based on the secondary batteries is considered the energy storage and conversion solution, with broad applicability in the new energy systems [2]. Investigations focus on developing new secondary battery systems with low cost, high energy density, and long cycle life. The lithium-sulfur (Li-S) secondary battery, which has a theoretical specific capacity of up to 1675 mAh/g and a theoretical energy density of 2600 kW/kg, is considered as one of the most promising next-generation secondary battery systems and has attracted extensive attention [3,4]. However, the shuttle effect of lithium polysulfides (LiPSs) between lithium metal anode and sulfur cathode results in serious problems such as the decrease of battery capacity, low coulombic efficiency, and the deterioration of cycle stability, which limit the practical application of Li-S batteries [5,6]. To overcome these issues, introducing a separator layer as a polysulfide shuttle barrier between anode and cathode is considered as an extremely effective strategy.

In recent years, researchers have proposed many approaches to optimize the construction of the separator layers to improve the battery’s performance by enhancing the conductivity and boosting the adsorption of LiPSs. Manthiram et al. [7,8,9,10] proposed a strategy of modifying the conductive layer on the cathode side of the electrode separator to improve the electrochemical reaction activity of the cathode active material, reducing the interface resistance and physically limiting the shuttle of polysulfides, thereby improving the battery rate performance and energy. Furthermore, Al_2_O_3_ [11,12], SnO_2_ [13], MnO_2_ [14], and other inorganic polar materials have been used to modify the conductive layer on the cathode side of the separator to improve the sulfur fixation effect of the separator. For example, N [15,16], P [17], O [18], B [19], and S [20,21] were also used to dope the carbon-based conductive layer on the cathode side of the separator to change the charge distribution state on the surface and improve the sulfur fixation. On this basis, the use of catalytic materials, which can capture and improve the conversion efficiency of LiPSs intermediates to functionalize the separator to improve the electrochemical performance of the battery, is an extremely effective strategy. Yi et al. [22] used Ir nanoparticles to modify KB to prepare the KB@Ir composite and used them as a catalytic layer on the separator to promote the redox reaction of lithium sulfide intermediates. The nanoparticles exhibited strong chemical adsorption on the polysulfide ions and effectively accelerated the kinetic process of polysulfide conversion. Giebeler et al. [23] embedded RuO_2_ into mesoporous carbon and coated it on the separator as an electrochemically active polysulfide nest, which significantly improved the redox reaction efficiency of the polysulfide that migrated out of the cathode. This type of multifunctional separator could have three advantages: (1) improving the electronic conductivity of the battery and the utilization rate of the active materials, (2) limiting the shuttle effect and reducing the dissolution and diffusion of polysulfides, and (3) accelerating the reaction kinetic process of polysulfides and the thermodynamic process of the solvent interface. However, this catalytic layer is mainly made of precious metals such as Au, Pt, Ir, and Ru or their oxides, and the high cost of these materials could limit their broad applicability [24]. Therefore, an optimization strategy for the low-cost separator that also has conductive, adsorption, and catalytic functions could have a significant influence on promoting the application of Li-S batteries.

Ketjen Black(KB) is a common commercial porous carbonaceous material with excellent conductivity, often used as the sulfur host [25,26] or the separator sulfur fixation body [27,28,29] of Li-S batteries to improve their conductivity. The fluffy accumulation of particles retains many pores, providing pathways for ion transfer. Moreover, its soft texture can alleviate the volume expansion of the cathode and separator. Meanwhile, in previous studies, NiFe_2_O_4_ mainly was used as a lithium storage material as the negative electrode material for lithium-ion batteries [30,31,32,33,34,35,36,37]. Recent studies have shown that nano NiFe_2_O_4_, which is mainly used as a lithium storage material for lithium-ion batteries, has a strong adsorption effect on polysulfides and capture-soluble polysulfides. Some researchers used it as a sulfur host and successfully inhibited the shuttle effect of polysulfides [38,39]. It is worth mentioning that NiFe_2_O_4_ has also been proven to accelerate the reaction kinetic process of polysulfides, which indicates that it has a relatively good catalytic effect on the redox reaction of polysulfides on the cathode [40,41,42]. Moreover, the modification of the positive electrode material is an efficient method to improve the electrochemical performance of Li-S batteries. Transferring the conductive carbonaceous material introduced into the cathode and the adsorptive catalytic material to the battery separator could be a promising strategy to enhance the development of Li-S batteries [43,44,45,46].

In this work, we innovatively loaded nano-NiFe_2_O_4_ on the membrane as a separator layer. The NiFe_2_O_4_/KB modified battery separator not only enhanced the conductivity of the separator but also improved its adsorption of polysulfides, accelerated the kinetic processes of oxidation and reduction of sulfides, and added the function of the secondary current collector to the separator. The results showed that the Li-S battery with the NiFe_2_O_4_/KB-modified separator had excellent rate performance and long-cycle stability. It maintained a discharge capacity of 406.27 mAh/g after 1000 stable cycles at a high current density of 1 C, with an average capacity attenuation of only 0.051% per cycle. This simple and effective method significantly improved the application capacity of Li-S batteries.

## 2. Materials and Methods

### 2.1. Preparation of NiFe_2_O_4_ Nanoparticles

NiFe_2_O_4_ nanoparticles were prepared by the improved hydrothermal method. Weigh 8 g of iron nitrate (Fe(NO_3_)_3_·9H_2_O (Aladdin Inc., Shanghai, China)), 2.9 g of nickel nitrate (Ni(NO_3_)_2_·6H_2_O (Aladdin Inc., Shanghai, China)), and 0.9 g of urea (CN_2_H_4_O (Aladdin Inc., Shanghai, China)) in 100 mL of deionized water and stir well, transfer to a hydrothermal autoclave and keep reacting at 180 °C for 24 h, then cool naturally to room temperature. After centrifugal collection of the precipitate, the precipitate was washed with water and ethanol, respectively, and the precipitation was dried at 60 °C for 12 h to obtain a reddish-brown powder. The dried powder was put into a tube furnace for heat treatment at 400 °C for 3 h and then ground uniformly to obtain NiFe_2_O_4_ nanoparticles.

### 2.2. Preparation of NiFe_2_O_4_/KB-Modified Separator

The ratio of the slurry and the orientation of the coating were determined based on the preliminary study in S1. The prepared NiFe_2_O_4_ nanoparticles, commercial KB, and binder PVDF were mixed to prepare a coating slurry with a mass ratio of 9:27:4. Firstly, the PVDF was dissolved into the 1-Methyl-2-pyrrolidinone (NMP) to obtain the mixed solution, and then the NiFe_2_O_4_ nanoparticles were added to the mixed solution by grinding and mixing the NiFe_2_O_4_ nanoparticles with the KB particles, which was stirred for 8 h at room temperature to obtain the coating slurry. A 50 μm spatula was used to coat the mixed slurry on the PP separator. After drying at 40 °C for 12 h, it was cut and stored in a glove box. The same method was used to prepare the KB-coated separator. Figure S1c shows a schematic diagram of the preparation process of the NiFe_2_O_4_/KB-modified separator.

### 2.3. Characterization of Materials

A Cu-Kα radiation X-ray diffractometer (Rigaku Inc., Tokyo, Japan) was used for X-ray powder diffractometry (XRD) to verify the formation of the products. A Raman spectrometer (Horiba Inc., Paris, France) was used to analyze the crystal structure of the prepared samples. An X-ray photoelectron spectroscope (Thermo Inc., Waltham, MA, USA) recorded the samples’ X-ray photoelectron spectrum (XPS). A Fourier Infrared Spectrometer (Thermo Inc., Waltham, MA, USA) was used to record the Fourier Transform Infrared (FT-IR) spectrum (S2) of the samples. The SEM micrographs were collected using a field emission environmental scanning electron microscope (FEI Inc., Hillsboro, OR, USA) equipped with an EDX microanalyzer. A field emission high-resolution projection electron microscope (FEI Inc., Hillsboro, OR, USA) was used to characterize the morphology of the prepared samples (S3).

### 2.4. Electrochemical Characterization

The assembled battery (S4) was left for 12 h to ensure that the electrolyte thoroughly wetted the inside of the battery. A Xinwei tester was used to conduct the constant current charge, and discharge tests of the battery were conducted at 1.7–2.8 V. An electrochemical workstation (Chenhua Inc., Shanghai, China) was used for cyclic voltammetry (CV) and electrochemical AC impedance (EIS) tests. The CV test voltage range was 1.5–3.0 V, and the sweep speed was 0.1 mV/s. The AC impedance scanning frequency was from the high frequency of 100 kHz to the low frequency of 0.01 Hz, with an amplitude of 5 mV.

### 2.5. Theoretical Calculation

The Vienna Ab initio Simulation Package (VASP) software was used for density functional theory (DFT) in the simulation calculations [47,48]. A 3 × 3 super-monolithic flat plate model containing two layers of atoms totaling 252 atoms was built to simulate the surface of NiFe_2_O_4_(1 0 0). The height of the vacuum layer was set to 15 Å. The cut-off energy was set to 500 eV. The sampling of the Brillouin zone was geometrically optimized using the Monkhorst–Pack format of a (1 × 1 × 1) k-point grid. The generalized gradient approximation (GGA) and the Perdew, Burke, and Ernzerhof (PBE) functional were used to optimize the structures [49,50]. The binding energy (E_b_) of the adsorbate was defined as

E_b_ = E_total_ − E_slab_ − E_s_ (1)

where E_total_ represents the total energy of the adsorbent model, and E_slab_ and E_s_ are the energy of the corresponding bare board and free adsorbent, respectively.

## 3. Results

### 3.1. Characterization of Nano NiFe_2_O_4_ Materials

**Figure 1:**
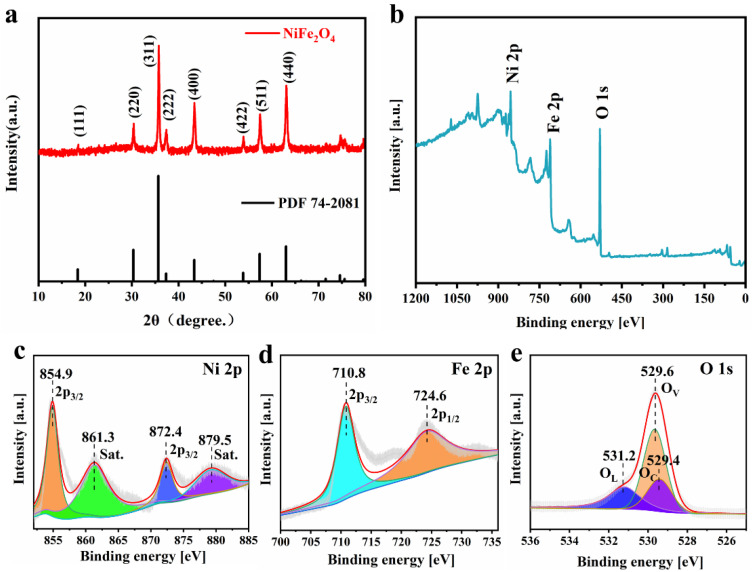
(**a**) The X-ray diffraction pattern of NiFe_2_O_4_, and the XPS spectra of NiFe_2_O_4_ nanoparticles: (**b**) survey spectra, (**c**) Ni 2p, (**d**) Fe 2p, and (**e**) O 1s.

Figure 1a shows the XRD pattern of the prepared NiFe_2_O_4_ sample. The characteristic peaks are assigned to (111), (220), (311), (222), (400), (422), (511), and (440) crystal planes. All the peak values correspond to the standard pattern of NiFe_2_O_4_ crystal (PDF 74-2801) [51]. There are no pronounced impurity peaks in the XRD pattern, and the peak shapes of all characteristic peaks are sharp, indicating that the crystallinity of the as-prepared NiFe_2_O_4_ material is high. The grain size of NiFe_2_O_4_ calculated based on the XRD pattern (S5) is 17–26 nm, with an average grain size of 20 nm. The NiFe_2_O_4_ nanoparticles can shorten the diffusion path of Li^+^ and expand the specific surface area to form more reaction sites.

XPS was used to obtain the detailed elemental composition and oxidation state characterization of the samples. Figure 1b shows the presence of three elements—Ni, Fe, and O—in the synthesized materials. The two prominent peaks in Figure 1c are located at 854.9 eV and 872.4 eV, corresponding to Ni 2p_3/2_ and Ni 2p_1/2_, respectively. In addition, the two satellite peaks of Ni^2+^ are located at 861.36 eV and 879.5 eV. The two main peaks at 710.85 eV and 724.65 eV in Figure 1d represent Fe 2p_3/2_ and Fe 2p_1/2_ of Fe^3+^. Figure 1e shows three peaks in the O 1s region, representing O_L_ (531.2 eV), O_C_ (529.4 eV), and O_V_ (529.65 eV), respectively [52].

The Raman spectrum (Figure S2a) shows that the NiFe_2_O_4_ nanoparticles have stable ferromagnetism, as the peaks at 219.12 cm^−1^, 285.88 cm^−1^, 479.68 cm^−1^, 572.4 cm^−1,^ and 689.93 cm^−1^ correspond to the spinel structure T_2g_(1), E_g_, T_2g_(2), T_2g_(3), and A1_g_ vibration, respectively [53,54]. No other impurity peaks are found on the Raman spectrum, implying that the crystal form of NiFe_2_O_4_ nanoparticles was uniform and consistent with the XRD results.

The FT-IR spectrum (Figure S2b) of the samples shows that there are two main absorption bands at 599.75 cm^−1^ and 472.47 cm^−1^, which correspond to the vibration of the tetrahedron and octahedron crystal structure of the NiFe_2_O_4_. The normal vibration mode of the tetrahedral cluster (599.75 cm^−1^) is higher than the vibration mode of octahedral clusters (472.47 cm^−1^). This is because the bond length of tetrahedral clusters is shorter than that of octahedral clusters, consistent with the previous reports [55]. The Raman spectrometer and infrared spectroscopy results are consistent with the XPS investigations, further confirming the synthesis of nano-NiFe_2_O_4_ with spinel structure.

**Figure 2:**
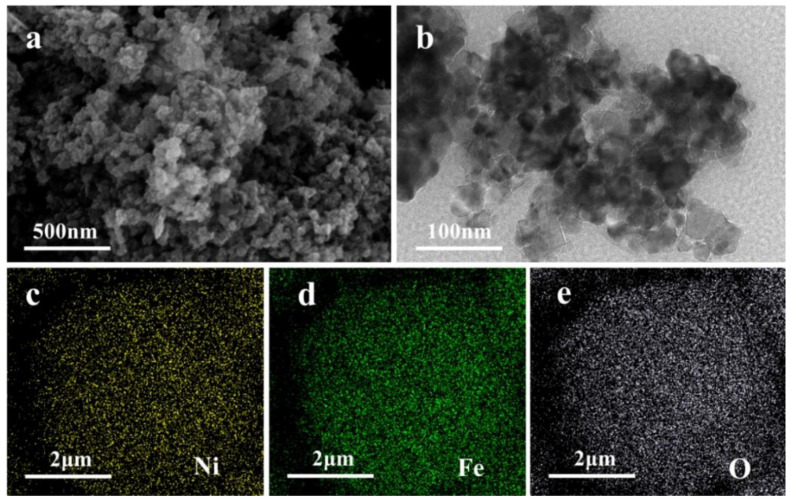
(**a**) The SEM and (**b**) TEM micrographs of NiFe_2_O_4_, and the element mapping of Ni (**c**), Fe (**d**), and O (**e**).

Figure 2a shows the SEM micrograph of the NiFe_2_O_4_ nanoparticles. It can be observed the particles have a very rough surface and a fluffy structure formed by cross-linking between particles. Figure S3a shows the N_2_ adsorption-desorption isotherms of NiFe_2_O_4_ nanoparticles. The Brunauer–Emmett–Teller method (BET) surface area of the NiFe_2_O_4_ nanocomposite was calculated to be 49.4 m^2^g^−1^. Figure S3a shows a typical type IV curve and type H3 hysteresis loop, indicating a majority of mesopores [56]. The rough surface can provide a larger specific surface area to provide sufficient active sites for the adsorption and conversion of lithium polysulfides on the separator. TEM further disclosed the detailed morphology of the samples (Figure 2b Figure S3b,c). The grain distribution of NiFe_2_O_4_ was relatively uniform, with a particle size within 20–30 nm, consistent with the XRD results. The EDS mapping results of O, Fe, and Ni elements (Figure 2c–e) reveal that the atomic ratio Ni content is 19% and Fe content is 40%. Their ratio is 1:2.1, which follows the ratio setting during the material preparation, and all of these are evenly distributed.

### 3.2. Morphology Analysis of NiFe_2_O_4_/KB-Modified Separator

**Figure 3:**
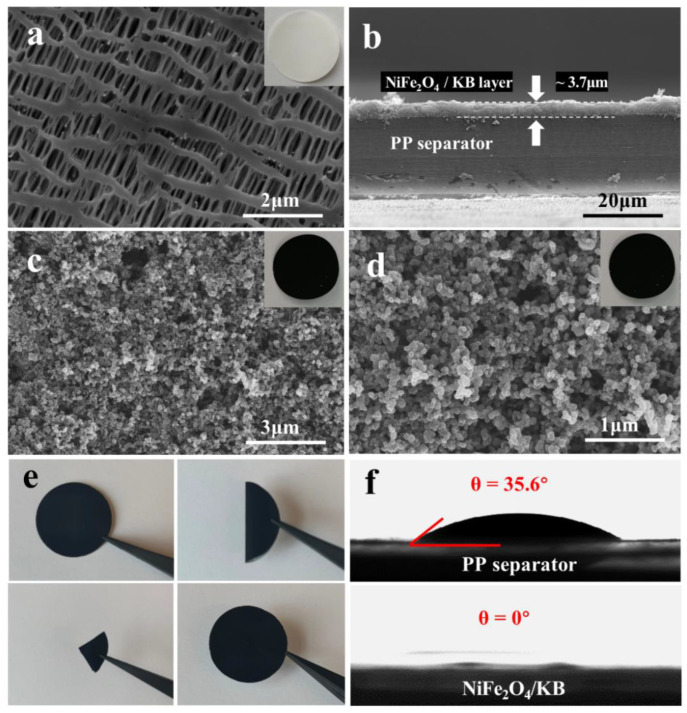
The SEM images of (**a**) the pristine PP separator and (**b**) the NiFe_2_O_4_/KB-modified separator in cross-sectional view, and (**c**,**d**) with different magnifications; (**e**) a digital photo of the NiFe_2_O_4_/KB-modified separator under mechanical stability tests; (**f**) the electrolyte contact angles of pristine PP and the NiFe_2_O_4_/KB-modified separator.

Figure 3a shows the SEM image of the pristine PP separator. The typical sub-micron long needle-like pores formed by the dry stretching process can be observed. These pores provide channels for the electrolyte penetration and lithium-ion transfer. However, the long-chain soluble LiPSs could easily pass through these large pores of the separator to the other side of the electrode, causing irreversible capacity loss.

In order to solve the shortcomings of the pristine PP separator without affecting the ion transmission, a modified layer with NiFe_2_O_4_/KB was coated on one side of the separator. Figure 3b shows a cross-sectional view of the NiFe_2_O_4_/KB-modified separator. The pristine separator of the coating layer was in close contact with the surface and adhered well. An appropriate thickness of about 3.7 μm was chosen to avoid the impact on the lithium-ion transmission and battery internal resistance from an overly thick coating. Figure 3c,d present the surface morphology of the NiFe_2_O_4_/KB-modified separator, and the upper right corner of Figure 3c shows the digital camera image of the as-coated modified separator. The SEM images show that the accumulated KB particles on the coating surface have a porous structure filled with liquid electrolytes, and convenient ion diffusion. NiFe_2_O_4_ nanoparticles are uniformly dispersed in the 3D porous structure formed by the accumulation of KB particles, which could anchor the polysulfide diffused from the cathode to the anode side. The accumulation formed the 3D structure, providing sufficient reaction space for the catalytic conversion. In addition, the functional carbon coating could act as a conductive secondary current collector, promoting electron transport and increasing the sulfur utilization rate. The two cooperate to firmly block the polysulfide on one side of the separator and prevent its shuttle effect on both sides of the separator, which becomes a powerful barrier to block the shuttle of polysulfide.

Figure 3e shows the folding/unfolding test of the modified separator. After repeated folding and deep bending, the modified material still adhered to the surface of the PP separator without any peeling. The results show that NiFe_2_O_4_/KB has good adhesion to the PP separator, and the modified separator has excellent mechanical stability and flexibility.

The wettability of the battery separator surface is an essential factor in improving interface compatibility, shortening the electrolyte filling time, and promoting lithium ions’ migration. The contact angle test was used to evaluate the wettability of these membranes. Figure 3f shows the contact angle of the electrolyte drop on the surface of the separator, and the contact angle between the electrolyte and the PP separator was 35.6°. When the electrolyte drop reached the surface of the NiFe_2_O_4_/KB separator, it immediately wet the NiFe_2_O_4_/KB separator. These results indicate that the NiFe_2_O_4_/KB coating could be beneficial to accelerate the penetration of the electrolyte, promoting the transmission of lithium ions and improving the electrochemical performance during the discharge/charge process.

### 3.3. Electrochemical Analysis of NiFe_2_O_4_/KB-Modified Separator

**Figure 4:**
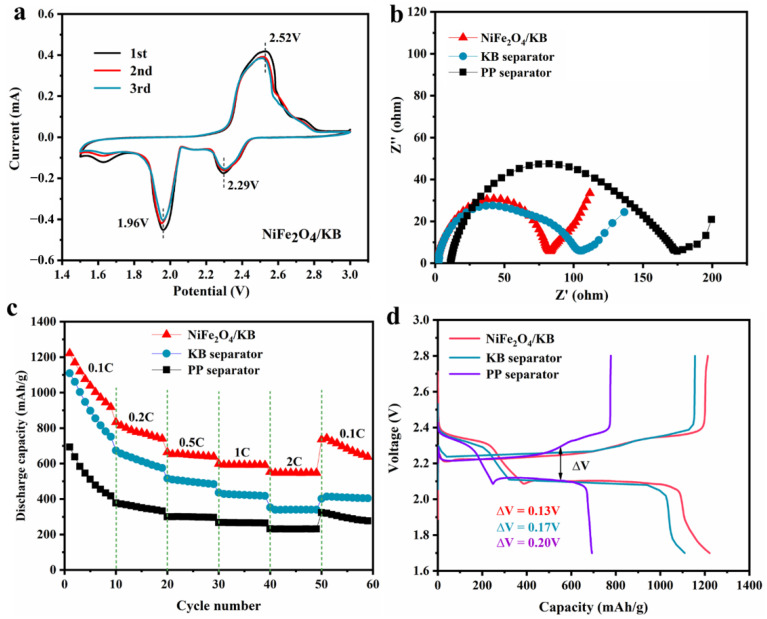
(**a**) The CV curves of the Li-S battery with the NiFe_2_O_4_/KB-modified separator; (**b**) the EIS of NiFe_2_O_4_/KB, KB, and the pristine PP separator; (**c**) the rate performance of the batteries with different separators between 0.1 C and 2 C; and (**d**) the charge/discharge curves of different separators at 0.1 C.

Figure 4 shows the electrochemical performance of the assembled Li-S batteries. The cyclic voltammetry (CV) curves of pristine PP separator (Figure S4a), KB modified separator (Figure S4b), and NiFe_2_O_4_/KB-modified separator batteries were taken in a voltage range of 1.5–3.0 V and a sweep rate of 0.1 mV/s. Figure 4a presents the CV curves of the first three circles of the NiFe_2_O_4_/KB-modified separator. The CV curves show two reduction peaks and a broad oxidation peak. The two reduction peaks at 2.29 V and 1.96 V are attributed to the reduction of S_8_ to lithium polysulfide (Li_2_S_x_, x ≥ 4) and further reduction to solid Li_2_S_2_ and Li_2_S. The broad oxidation peak near 2.52 V was attributed to the coupling conversion of Li_2_S_2_/Li_2_S to LiS_8_/S [10,57,58,59]. The peak voltage in the CV curves does not change significantly, indicating that the NiFe_2_O_4_/KB-modified separator has good redox reversibility. Figure 4b shows the EIS of the NiFe_2_O_4_/KB-modified separator. The semicircular high-frequency area of the EIS diagram represents the charge transfer resistance (*R*_ct_) of the electrochemical reaction at the electrode interface, and the oblique line low-frequency area presents the Warburg impedance related to the ion diffusion in the electrolyte [60,61,62]. The EIS diagram shows that the charge transfer resistance of the NiFe_2_O_4_/KB-modified separator (*R*_ct_ = 80.39 Ω) is lower than that of the PP (*R*_ct_ = 184.2 Ω) and KB (*R*_ct_ = 110.3 Ω) separators. Table S1 shows the ionic conductivity data calculated from the EIS for different separators. There was no increase in the battery’s internal resistance due to the addition of the coating layer, revealing that the introduction of the coating layer increases the interface conductivity and enhances the transfer of interface charges. The NiFe_2_O_4_ on the separator provides the active site, which strongly interacts with the soluble polysulfide and greatly accelerates the kinetics of the redox reaction, which is consistent with the better rate performance of the battery [63].

Figure 4c shows the rate performance of these batteries with different separators. It can be observed that the rate performance of the NiFe_2_O_4_/KB-modified separator assembled battery is significantly better compared to other batteries. At rates of 0.1, 0.2, 0.5, 1, and 2 C, the discharge capacities of the NiFe_2_O_4_/KB-modified separator assembled battery are 1221.4, 833.1, 663.6, 598.1, and 553.2 mAh/g, respectively. When the rate is restored to 0.1 C, the battery capacity is 735.8 mAh/g. When the KB separator is used, the capacities are 1109.7, 673.6, 516.3, 435.7, and 352.8 mAh/g, respectively. Furthermore, when the rate is restored to 0.1 C, the battery capacity is 401.5 mAh/g. The battery capacities of the pristine separator are 692.8, 377.6, 300.2, 267.2, and 232.2 mAh/g, respectively, and when the rate is restored to 0.1 C, it is 323.1 mAh/g. Figure S5 shows the charge/discharge profiles of the corresponding separators at different rates. Figure 4d shows the charge/discharge profiles of three batteries at 0.1 C, and two distinctive discharge platforms of Li-S batteries are observed. The short discharge platform at a high potential of 2.3 V corresponds to converting elemental S_8_ into soluble long-chain polysulfide LiPSs, and the low potential at 2.1 V corresponds to the process of converting LiPSs into insoluble discharge end products Li_2_S_2_/Li_2_S [64]. For the pristine PP separator, KB-modified separator, and NiFe_2_O_4_/KB-modified separator, the potential intervals between the charge and discharge platforms were 0.13, 0.17, and 0.20 mV, respectively. Compared with the PP and KB-modified separators, the NiFe_2_O_4_/KB-modified separator has the smallest potential interval, indicating that the NiFe_2_O_4_/KB-modified separator battery has better dynamic characteristics.

**Figure 5:**
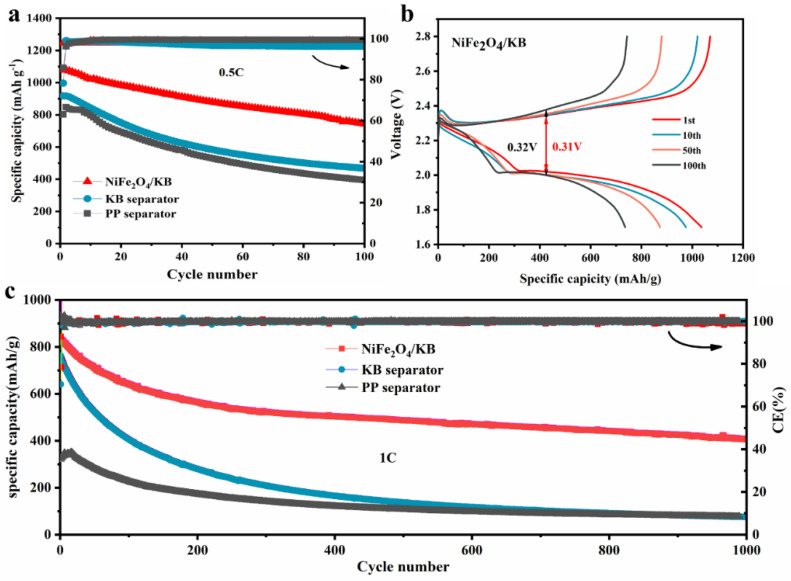
(**a**) The cycling performance of different separators at 0.5 C (the first two circles at 0.1 C), (**b**) the charge/discharge curves with different cycles of NiFe_2_O_4_/KB-modified separator battery at a current density of 0.5 C, and (**c**) the long-term cycling stability of separators at 1 C.

Figure 5 shows the cycling performance of batteries with different separators. The batteries assembled with different separators were subjected to the long-cycle tests at 0.5 C and 1 C, respectively. Figure 5a shows the cycling performance graph of batteries equipped with different separators at 0.5 C. The initial discharge capacity of the battery with the pristine separator is 802.6 mAh/g and decreases to 394.6 mAh/g after 100 cycles, and the retention rate is only 49.1%. However, when the separator is modified with a pure KB coating layer, the capacity retention rate is 52.3%, a significant improvement compared with the pristine separator (the initial capacity of this battery is 917.6 mAh/g and decays to 479.9 mAh/g after 100 cycles). It can be observed that the improvement of the stability is limiting when only carbon layer is added. However, the first capacity reaches as high as 1079.6 mAh/g and remains at 753.1 mAh/g after 100 cycles (with a capacity retention rate of 69.7%) with the NiFe_2_O_4_/KB-modified separator. It can be observed that the battery capacity retention rate of the NiFe_2_O_4_/KB-modified separator is about 1.5 times that of the pristine separator, which exhibits good cycling performance. This may be attributed to the improved conductivity due to the introducing of KB nanoparticles. Furthermore, the chemical adsorption of NiFe_2_O_4_ particles relieves the shuttle effect of polysulfides, improves the dynamic conversion rate of polysulfides, and increases the utilization rate of active materials. Moreover, the coating layer has good hydrophilicity and liquid retention to store more electrolytes and improved ion conductivity.

Figure 5b shows the charge/discharge curves using the NiFe_2_O_4_/KB-modified separator at a current density of 0.5 C with different cycling numbers. The average overpotential of the battery with the original PP separator increases from 0.18 V to 0.37 V (Figure S6a) after 100 cycles. In comparison, the average overpotential of the KB-modified separator battery rises from 0.20 V to 0.34 V (Figure S6b). On the other hand, the overpotential of the battery with NiFe_2_O_4_/KB-modified separator has no noticeable change, and the capacity decay rate is slower. Studies have proved that pure KB has a particular effect on the modification of the separator [26,29]. However, the effect is more evident after adding NiFe_2_O_4_, indicating that the addition of NiFe_2_O_4_ plays a crucial role in improving the electrochemical performance of Li-S batteries.

Figure 5c shows the cycling performance graph of NiFe_2_O_4_/KB-modified separator at a high rate. The corresponding charge/discharge platform (Figure S7) shows that the NiFe_2_O_4_/KB-modified separator battery has the smallest overpotential (0.29 V) at a high rate. Furthermore, the NiFe_2_O_4_/KB-modified separator battery still has a relatively high initial discharge capacity at a high current density of 1 C. At the high current density, its decay rate is faster than that of the low density. After 100 cycles, the battery capacity decreases to 641.2 mAh/g. The battery still has a specific discharge capacity of 406.27 mAh/g after 1000 cycles, with an average capacity attenuation of only 0.051% per cycle. This stable cycling performance is better compared with previous results (Table S2). The accelerated decay rate can occur when the current density is high; the formation rate of lithium polysulfide is fast; and the output is large, that is, higher than the adsorption capacity of NiFe_2_O_4_ particles. It can be seen that compared to the battery with the NiFe_2_O_4_/KB-modified separator, the batteries with the pristine separator and the KB-coated separator have a very rapid capacity decay during the long 1000 cycles. Therefore, it can be concluded that using NiFe_2_O_4_/KB to coat the separator can significantly improve the battery’s cycle life and make the battery cycle stable for a long time.

**Figure 6:**
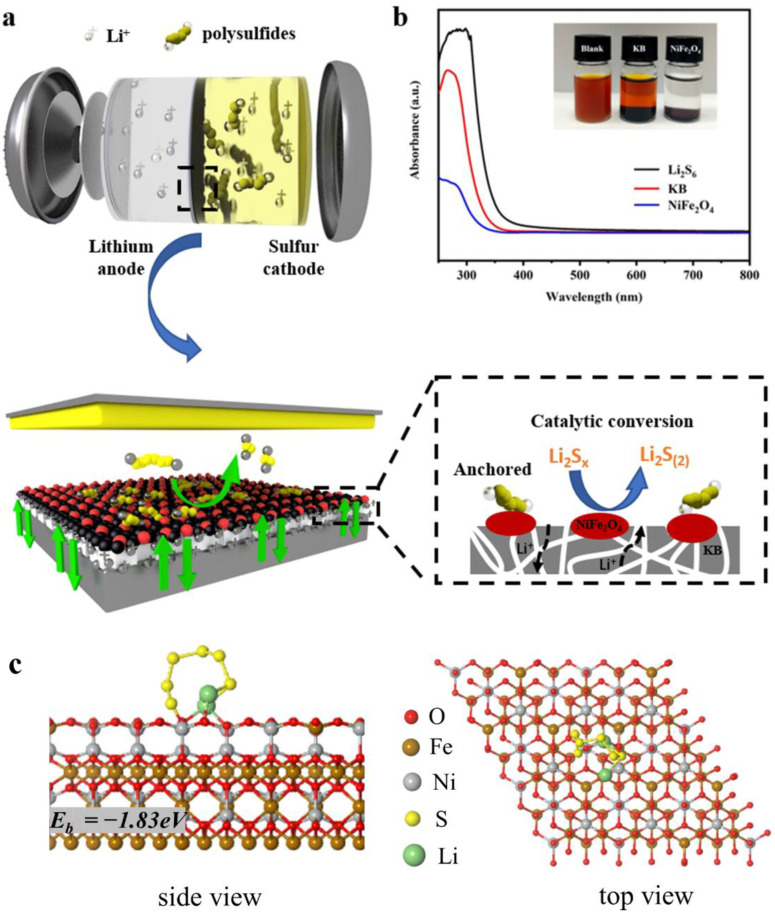
(**a**) The working principle of the NiFe_2_O_4_/KB-modified separator Li-S battery; (**b**) the UV-Visible spectrum of the Li_2_S_6_ solution containing KB and NiFe_2_O_4_ (the inset is a photo of sealed vials of Li_2_S_6_/DOL/DME solutions after contact with KB and NiFe_2_O_4_ 1 h later), and (**c**) the side view of and top view of the adsorption configuration of Li_2_S_6_ on the NiFe_2_O_4_ (1 0 0) surface.

Figure 6a shows the working principle diagram of a Li-S battery with a NiFe_2_O_4_/KB-modified separator. The soluble polysulfide ions from the desulfurized cathode could form S_8_, and the short-chain insoluble Li_2_S_2_ and Li_2_S were deposited on the separator through the disproportionation reaction. Since the separator is an electronic insulator, the “dead sulfur” deposited on the separator can no longer participate in the electrochemical reaction, resulting in the decrease of the battery’s energy density. Under the premise of ensuring the electronic insulation between the positive and negative electrodes, KB was introduced as a conductive layer between the separator and the cathode, and the fluffy KB particles were piled together. On the one hand, it could buffer the shuttle of polysulfide ions in the cathode and has a certain physical inhibitory effect on the migration of polysulfides, slowing down the attenuation of battery capacity. On the other hand, the KB conductive layer could be used as a “second current collector”, providing a place for the electrochemical reaction of polysulfide ions. It could activate the “dead sulfur” to avoid the capacity loss caused by the deactivation of active materials.

The adsorption and catalytic effects of NiFe_2_O_4_ were the keys to achieving long-term stable cycling of Li-S batteries, which could be further verified by the polysulfide adsorption experiments (S13). It can be seen from Figure 6b that the Li_2_S_6_ solution became colorless after NiFe_2_O_4_ was added, while the color of the sample with KB remained nearly unchanged. This shows that NiFe_2_O_4_ has an excellent adsorption capacity for polysulfides. Further UV-Vis measurements showed that the intensity corresponding to the S_6_^2-^ peak at 280 nm for the sample with KB decreases slightly [65]. On the other hand, after the addition of NiFe_2_O_4_, the absorption intensity of the S_6_^2−^ peak decreases significantly, further verifying the excellent adsorption capacity of NiFe_2_O_4_ for polysulfides, which plays a key role in the long-term stable cycling of the battery.

According to density functional theory (DFT) calculations, the interactions between NiFe_2_O_4_ and polysulfides are deeply investigated, and the binding energy of Li_2_S_x_ (x = 1, 2, 4, 6) with NiFe_2_O_4_ is calculated. Figure 6c and Figure S8a show the adsorption configuration of Li_2_S_x_ (x = 1, 2, 4, 6) with the NiFe_2_O_4_ (1 0 0) surface in side and top view. DFT calculations show that the adsorption energy of NiFe_2_O_4_ for Li_2_S_6_ is −1.83 eV. In addition, calculations show that NiFe_2_O_4_ has strong adsorption for Li_2_S_6_, Li_2_S_2,_ and Li_2_S_4_ (Figure S8b). The high binding energy between polysulfide molecules and NiFe_2_O_4_ indicates that NiFe_2_O_4_ has a strong polysulfide limiting ability, which is also consistent with the results of the polysulfide adsorption tests. NiFe_2_O_4_ could form a strong adsorption effect for polysulfides by the formation of Li-O and S-O bonds. The strong adsorption between polysulfides and NiFe_2_O_4_ could eliminate the shuttle effect of polysulfides and contribute to the excellent cycling stability. Moreover, nano- NiFe_2_O_4_ could act as a catalyst for the conversion of polysulfides. The NiFe_2_O_4_/KB layer coating does not block the original separator’s pores, and the tiny NiFe_2_O_4_ nanoparticles have a larger specific surface area to form more reaction sites. The modified separator that combines NiFe_2_O_4_ and KB, on the one hand, could use the physical adsorption and barrier of KB and the chemical adsorption capacity of nickel ferrite to block polysulfides on the side of the cathode to prevent the shuttle of polysulfides. On the other hand, NiFe_2_O_4_ can give full play to its catalytic effect on the “second current collector” formed by the KB conductive layer and improve polysulfides’ conversion efficiency. The synergy significantly reduces the shuttle effect of polysulfides, which is important for reducing the corrosion loss of lithium, enhances the rate electrochemical performance; and promotes the long-term stability of the Li-S battery.

## 4. Conclusions

This section is not mandatory but can be added to the manuscript if the discussion is unusually long or complex. We prepared NiFe_2_O_4_ nanoparticles by a simple hydrothermal method and improved the wettability of the separator to the electrolyte using the good hydrophilicity of NiFe_2_O_4_. NiFe_2_O_4_ used its binding force toward polysulfides and chemical adsorption to hinder the shuttle effect. In order to avoid the introduced NiFe_2_O_4_ particles hindering the lithium-ion transmission channel, porous carbon KB was introduced to resolve this situation. The introduced carbon layer could construct a porous transmission channel, improve the interface conductivity of the separator and the cathode, increase the electron conductivity, restrict the polysulfide diffusion, and improve the transfer of lithium ions without avoid increasing the internal resistance of the battery. The NiFe_2_O_4_/KB-modified separator battery could reach a capacity of 1079.6 mAh/g at 0.5 C. After 100 cycles, the capacity remained at 753.1 mAh/g, and the capacity retention rate was 69.7%. After 1000 cycles at a high rate of 1 C, the battery still had a specific discharge capacity of 406.27 mAh/g, and the average capacity attenuation per cycle was only 0.051%. The experiments have proved that using the NiFe_2_O_4_/KB-surface-coating-modified separator is a simple and effective method to improve the performance of the Li-S battery.

## Data Availability

Data presented in this article are available on request from the corresponding author.

## Supplementary Materials

The following supporting information can be downloaded at: https://www.mdpi.com/article/10.3390/nano12081347/s1, Figure S1: schematic illustration of coated NiFe_2_O_4_/KB separator; Figure S2:(a) Raman spectra of NiFe_2_O_4_ nanocomposite;(b) FT-IR spectra of NiFe_2_O_4_ nanoparticles. Figure S3: N_2_ adsorption–desorption isotherms of NiFe_2_O_4_ nanoparticles; Figure S4: (a) cyclic voltammetry curves of the battery with PP separator; (b) cyclic voltammetry curves of the battery with KB separator. Figure S5: (a) charging and discharging curves of PP separator at different rates; (b) charging and discharging curves of KB separator at different rates; (c) charging and discharging curves of NiFe_2_O_4_/KB separator at different rates. Figure S6: charge/discharge voltage profiles with PP (a) and KB (b) separator at 0.5 C with different cycles. Figure S7: (a) charge/discharge voltage profiles with different separator at 1C for the first cycle; charge/discharge curves of cells with PP (b), KB (c), and NiFe_2_O_4_/KB separator (d) at 1C for different cycles. Figure S8: (a) side view of and top view of the adsorption configuration of Li_2_S_x_ (x = 1,2,4) on the NiFe_2_O_4_ (1 0 0) surface; (b) the adsorption energy of Li_2_S_x_ (x = 1,2,4,6) on the NiFe_2_O_4_ (1 0 0) surface. Table S1: summary of the electrochemical performance of the Li−S batteries configured with different modified separators and interlayers. References [66,67,68,69,70,71,72,73,74,75,76,77,78,79,80,81,82,83] are mentioned in Supplementary Materials.
