# Supplementary material for: NiFe2O4/Ketjen Black Composites as Efficient Membrane Separators to Suppress the Shuttle Effect for Long-Life Lithium-Sulfur Batteries
Source: Nanomaterials (Basel). 2022 Apr 14;12(8):1347. doi: 10.3390/nano12081347 (PMC9031026; doi:10.3390/nano12081347)
Supplement: Supplementary file 1 [file nanomaterials-12-01347-s001.zip › nanomaterials-1653499-supplementary.pdf]

Supporting information

$NiFe_2O_4$/Ketjen Black Composites as Efficient Membrane Separators to Suppress the Shuttle Effect for Long-Life Lithium-Sulfur Batteries

Wen Jiang [1], Lingling Dong [1], Shuanghui Liu [1], Shuangshuang Zhao [2], Kairu Han [1], Weimin Zhang [1], Kefeng Pan [1,*] and Lipeng Zhang [2,*]

[1] School of Chemistry and Chemical Engineering, Shandong University of Technology, Zibo 255049, China; wengejang@126.com (W.J.); donglingling202107@163.com (L.D.); shuanghuiliu2020@163.com (S.L.); hankairu2022@126.com (K.H.); wmzhang@sdut.edu.cn (W.Z.)

[2] School of Materials and New Energy, South China Normal University, Shanwei 516600, China; 20219207@m.scnu.edu.cn

* Correspondence: xiaopandy@126.com (K.P.); zhanglipeng@sdut.edu.cn (L.Z.)

S1 Preliminary study to determine the slurry ratios

The ratio of the slurry and the orientation of the coating were determined based on the preliminary study. Firstly, the pure $NiFe_2O_4$ nanoparticle separator ($NiFe_2O_4$: PVDF mass ratio of 9:1 Figure S1a) were prepared and assembled a lithium-sulfur battery for constant current charge and discharge tests. The test results were not satisfactory, the charge/discharge capacity of the battery was too low and the decay rate was fast, it is possible that the pure $NiFe_2O_4$ coating will block the separator and affect the ion transport. The orientation of the flipped separator (Inverted $NiFe_2O_4$) is used to study the effect of coating orientation on battery performance. The results show that the performance of the battery coated on the sulfur cathode side is better than the coating material on the side of the lithium anode. However, the performance of pure $NiFe_2O_4$ coated separator battery is very poor. Therefore, KB was considered to combine with $NiFe_2O_4$ to prepare a separator with an $NiFe_2O_4$: KB mass ratio of 1:1 to test the battery performance, which resulted in a significant improvement in battery performance (Fig S1b). The amount of $NiFe_2O_4$ was further reduced and a separator was prepared with a mass ratio of $NiFe_2O_4$: KB of 1:3 and a mass ratio of mixed material to binder PVDF of 9:1 (the mass ratio of $NiFe_2O_4$/KB/PVDF is 9:27:4), and assembled the battery for charge/discharge testing, and obtained more satisfactory results. Figure S1c shows a schematic diagram of the preparation process of the $NiFe_2O_4$/KB modified separator.

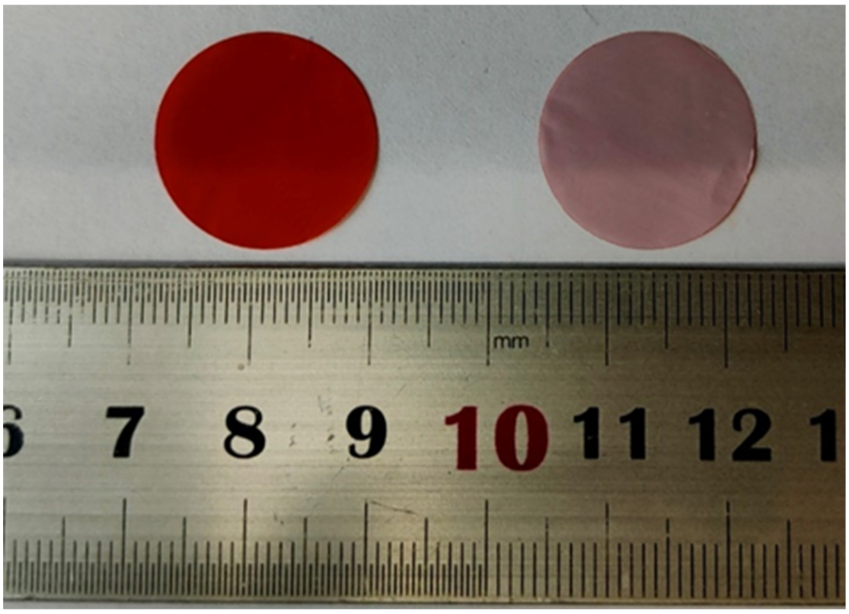

Figure S1(a) Digital photos of the front and back of pure $NiFe_2O_4$ nanoparticle coated separator.

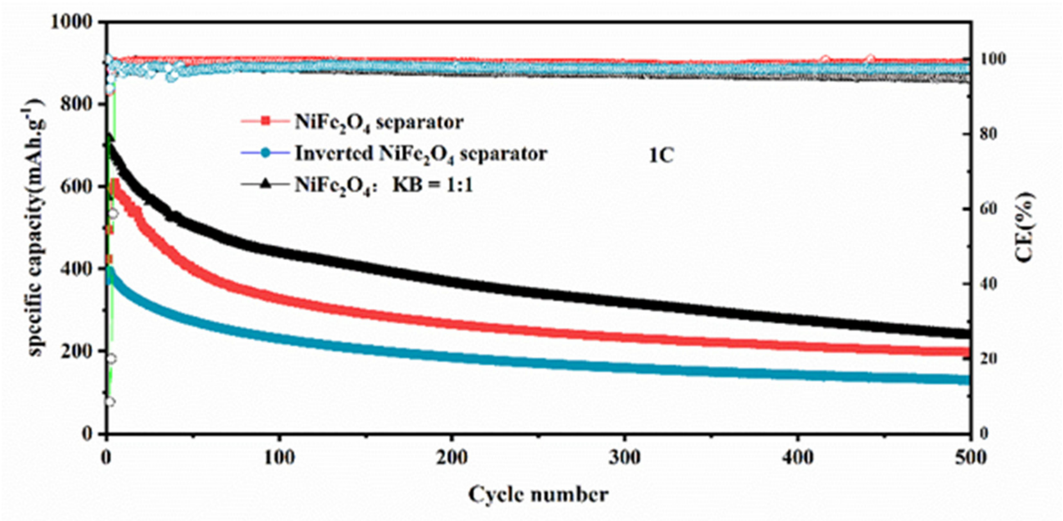

Figure S1(b) Long cycle test of lithium-sulfur batteries with different mass ratios and different assembly methods at 1C.

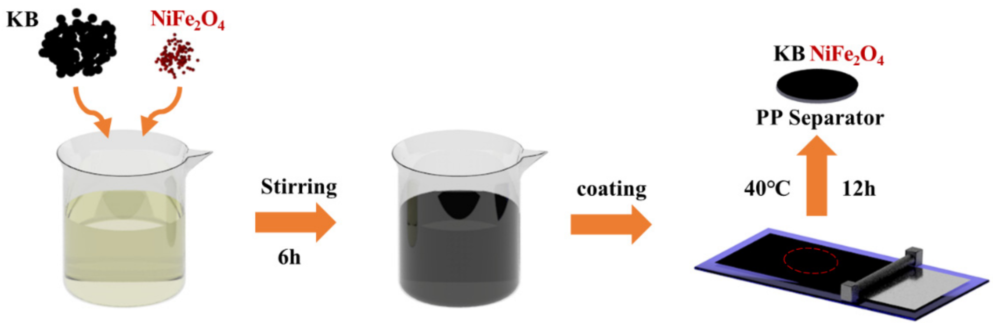

Figure S1(c) Schematic illustration of coated $NiFe_2O_4$/KB separator

S2 Material characterization

The XRD data were collected at room temperature with a test range of 10°–80° and a scan speed of 10°/min. The X-ray diffraction maps were indexed using the Joint Committee on Powder Diffraction Standards (JCPDS) card. The Raman spectrometer has an Ar ion wavelength of 785 nm, a power of 20 MW, and a sweep range of 50-3200 cm^{-1} (785 nm). The emission source of XPS is monochromatic Al-Kα rays with an energy of 1486.6 eV. Fourier transform infrared (FT-IR) spectra were recorded using a Fourier infrared spectrometer (Nicolet 5700), and FT-IR spectra in the range of 4000-400 cm were recorded once to study the nature of the formed chemical bonds.

S3 Sample preparation

The synthesized sample particles were well ground in a mortar and pestle and then sonicated for 30 min in a beaker containing anhydrous ethanol to disperse the samples. The sample was sucked onto the copper grid with a disposable dropper and added once or twice, and after the sample was completely dried, it was glued on the electron microscope stage with conductive adhesive for scanning electron microscope testing. Due to the ferromagnetic nature of the samples, double networking was used in the preparation of the projection electron microscope samples to prevent sample aspiration into the electron microscope chamber. For septum testing, the prepared septum is cut to the appropriate size and glued to the electron microscope stage with conductive adhesive for testing. The diaphragm sections were prepared using a special cross-

sectional electron microscope stage.

S4 Sulfur cathode fabrication and battery assembly

Sublimated sulfur, KB and binder (polyvinylidene fluoride, PVDF) were dissolved in N-methyl pyrrolidone in the mass ratio of 7:2:1, and the mixture was stirred for 12 h. After that, the coated poles were coated on the aluminum foil with a scraper, and the coated poles were dried in a vacuum drying oven at 60°C to remove the solvent, and cut into round poles with a cutting machine, and the sulfur loading of the poles was about 0.8-1.0mg/cm^2. The CR2032 Li-S button cell was assembled with $NiFe_2O_4$/KB, KB and commercial PP separators respectively in a glove box (H_2O<0.1ppm O_2<0.1ppm) under high purity argon atmosphere.

S5 Grain size calculation

The grain size was calculated by the Schell equation combined with XRD patterns.

$$D = \frac{K\lambda}{\beta \cos\theta}$$

Where D=crystallites size (nm)； K=0.9 (Scherrer constant) ； λ=0.15406 nm(wavelength of the x-ray sources) ； θ=Peak position (radians)

The calculated results show that the grain size of the synthesized $NiFe_2O_4$ nanoparticles is in the range of 17 nm–26 nm, with an average grain size of 20 nm. The micro-sized $NiFe_2O_4$ particles can shorten the diffusion path of Li^+ and expand the specific surface area to form more reaction sites.

S6 Raman Spectroscopy and FT-IR spectra

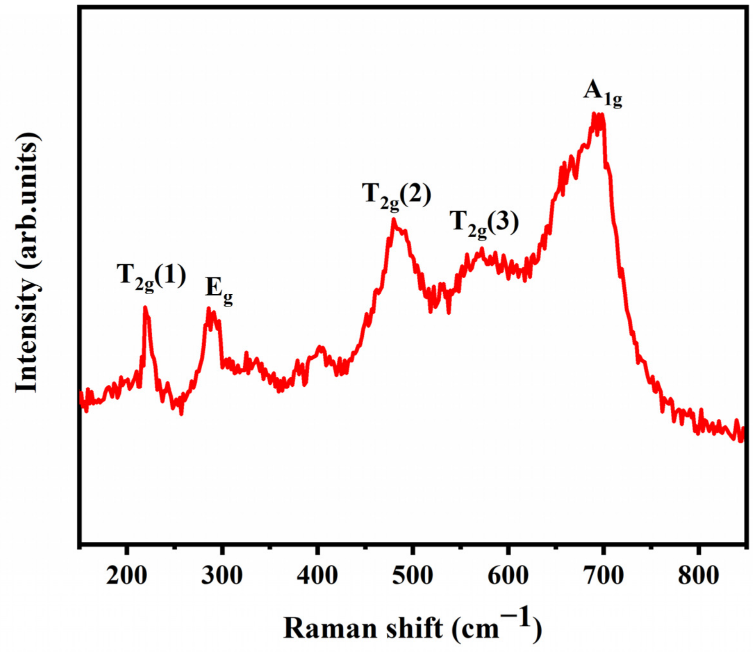

Figure S2 (a) Raman spectra of $NiFe_2O_4$ nanocomposite.

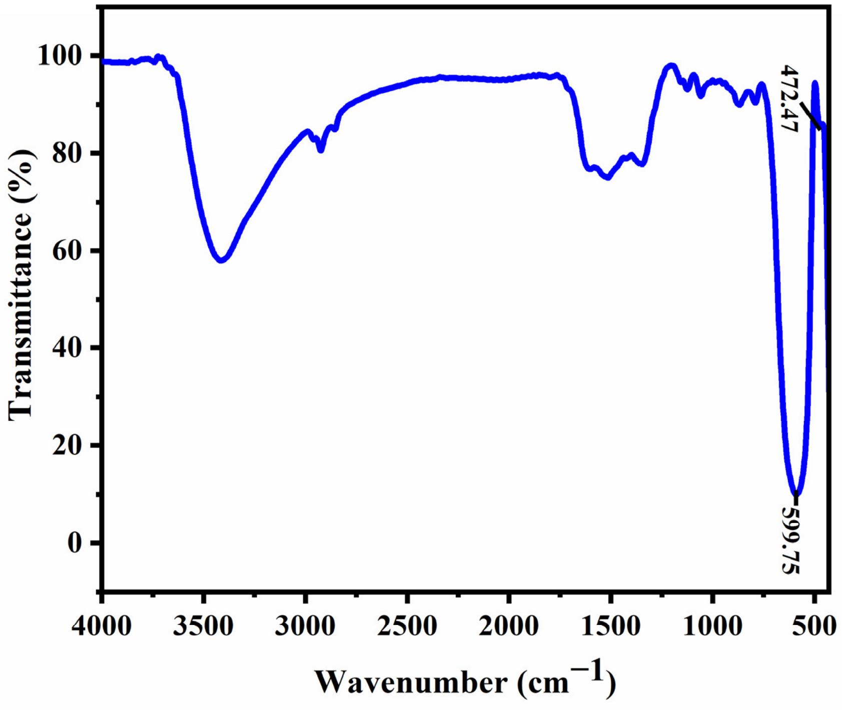

Figure S2 (b) FT-IR spectra of $NiFe_2O_4$ nanoparticles.

S7 N_2 adsorption–desorption isotherms and TEM of $NiFe_2O_4$ nanoparticles.

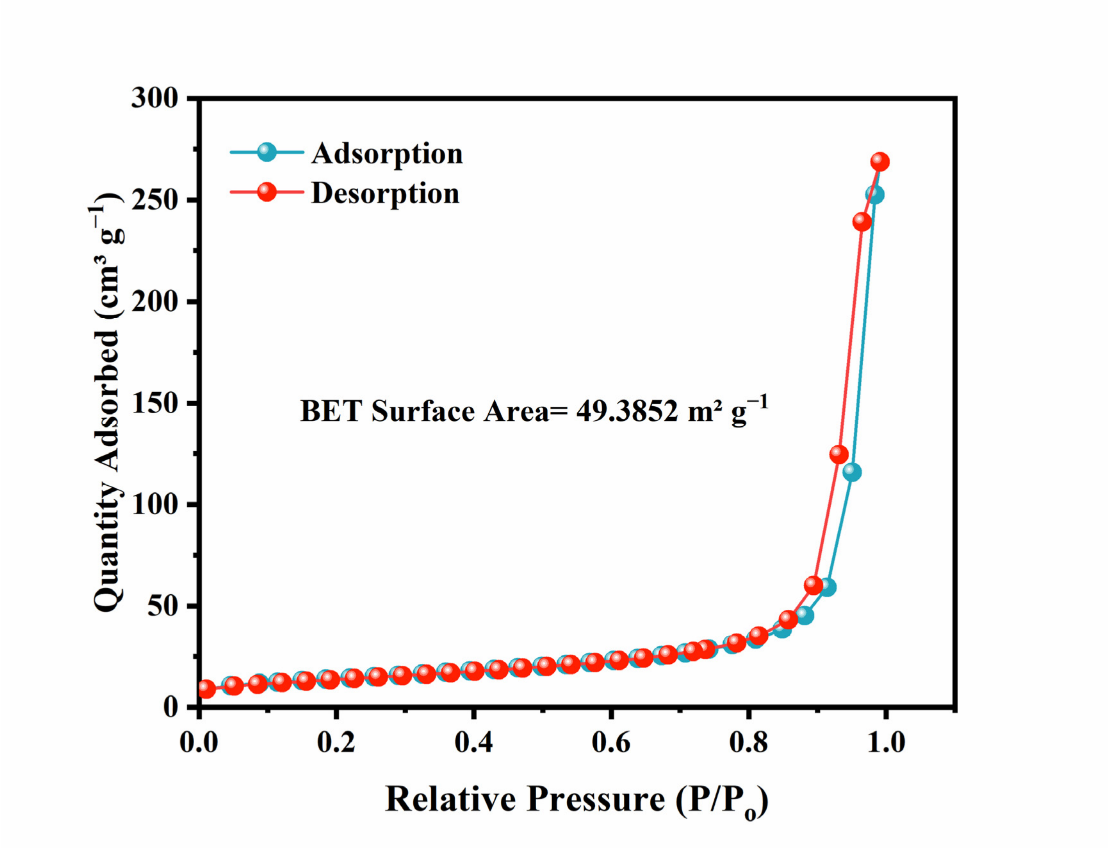

Figure S3 (a) N_2 adsorption-desorption isotherms of $NiFe_2O_4$ nanoparticles.

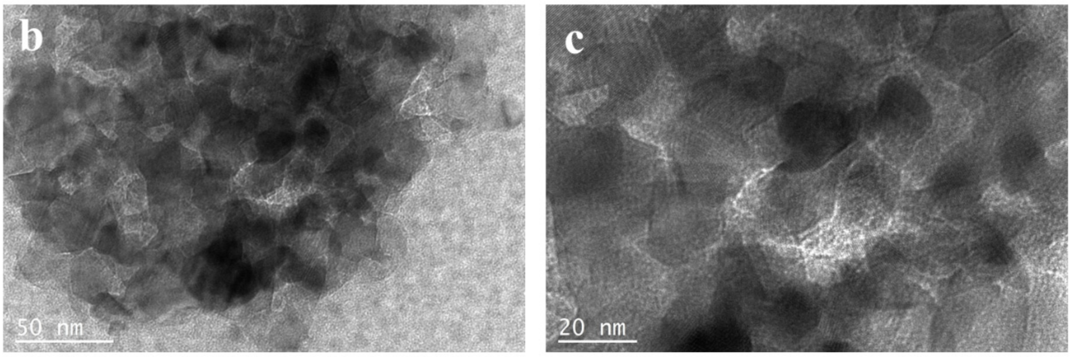

Figure S3 (b) and (c) TEM of the $NiFe_2O_4$ nanoparticles.

S8 The CV curves of the battery with KB and PP separator.

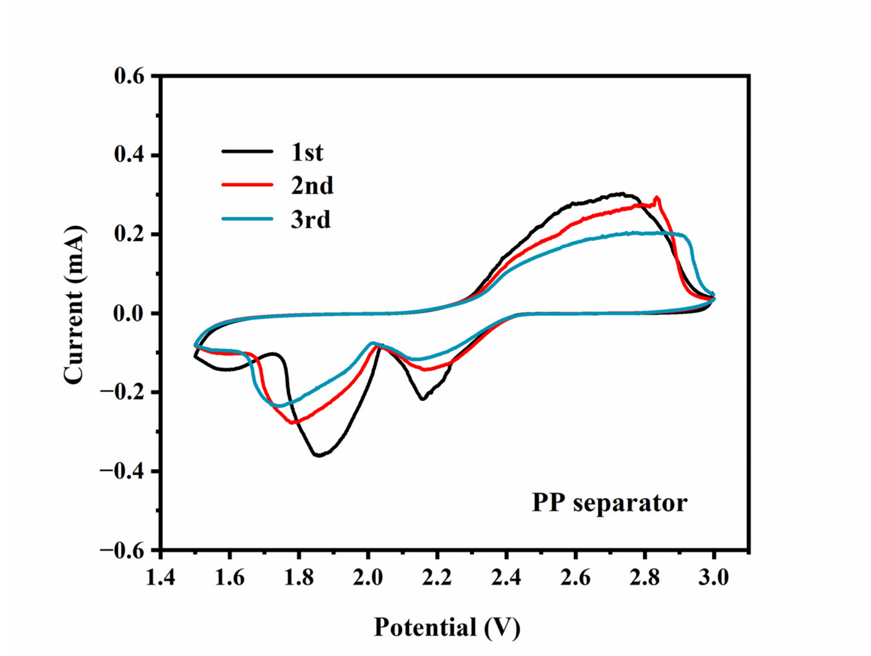

Figure S4 (a) Cyclic voltammetry curves of the battery with PP separator.

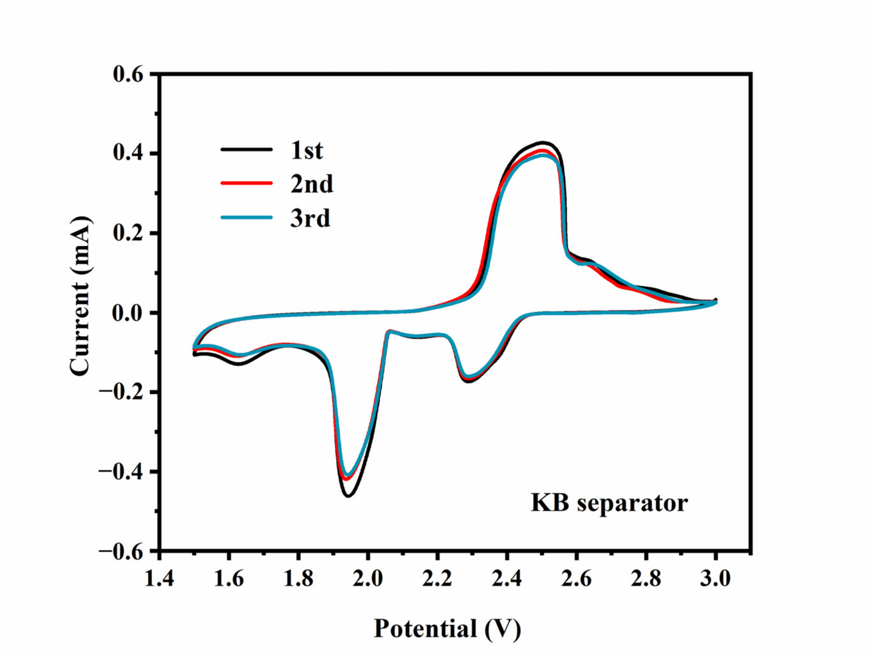

Figure S4 (b) Cyclic voltammetry curves of the battery with KB separator.

Table S1. Ionic conductivity of the different separators.

Sample	Ionic conductivity (mS cm^{-1})
PP separator	1.2
KB separator	2.3
$NiFe_2O_4$/KB separator	3.1

S9 Charge/discharge voltage profiles at different rate with different separators

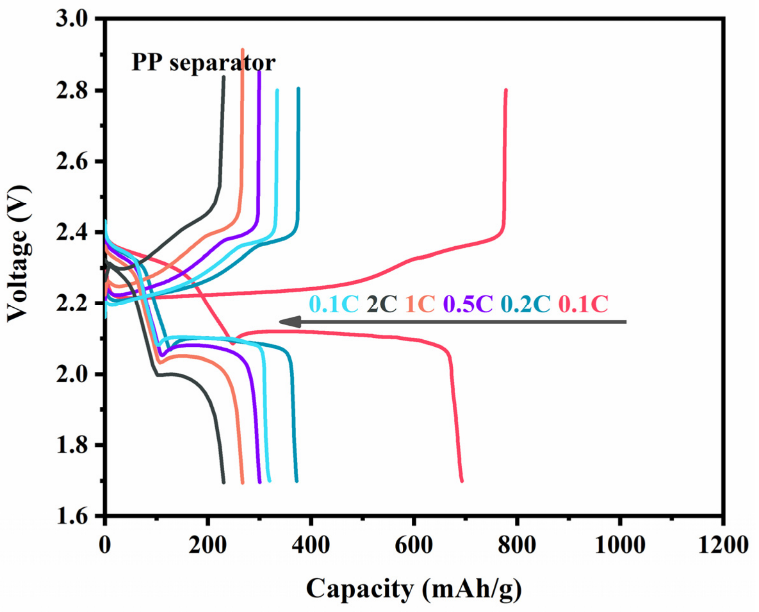

Figure S5 (a) Charging and discharging curves of PP separator at different rates.

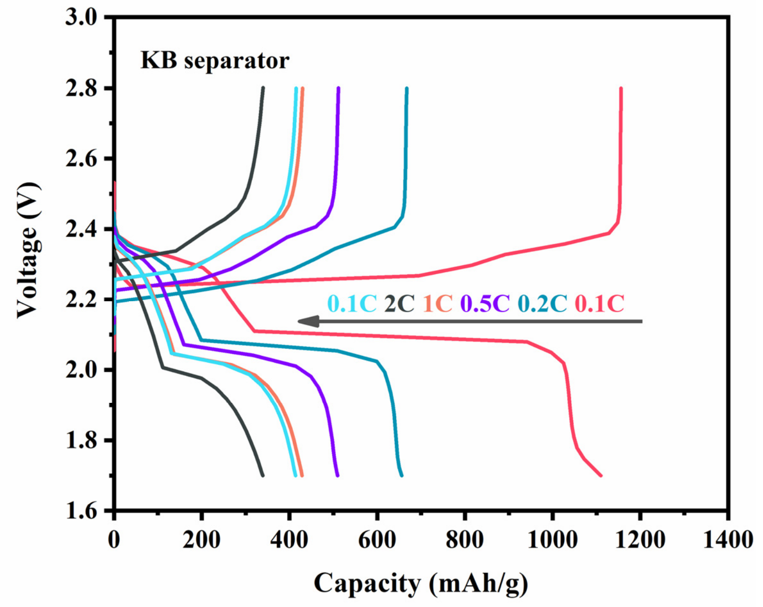

Figure S5 (b) Charging and discharging curves of KB separator at different rates.

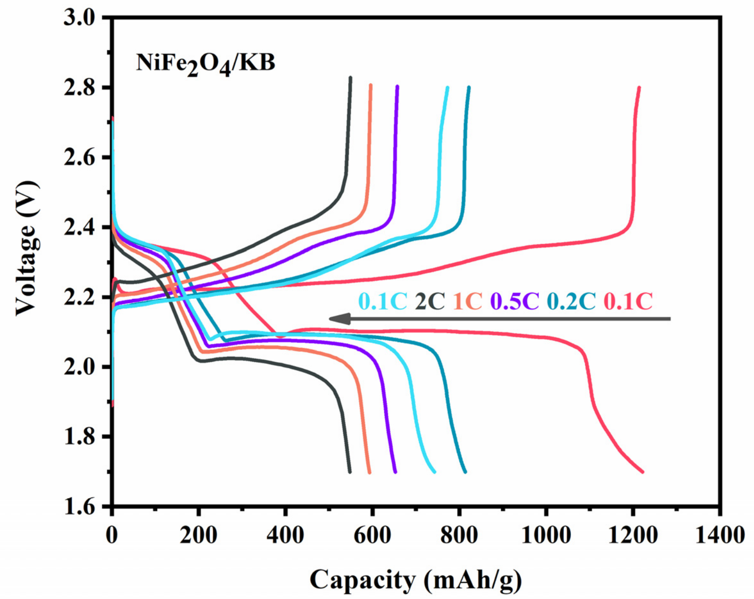

Figure S5 (c) Charging and discharging curves of $NiFe_2O_4$/KB separator at different rates.

S10 Charge/discharge voltage profiles with different separators at 0.5C with different cycles

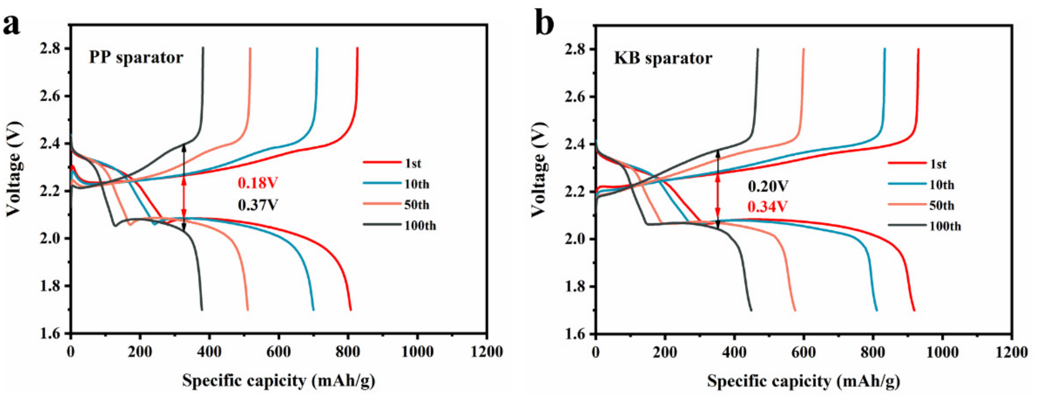

Figure S6 Charge/discharge voltage profiles with PP (a)and KB(b) separator at 0.5C with different cycles.

S11 Charge/discharge voltage profiles with different separators at 1C with different cycles

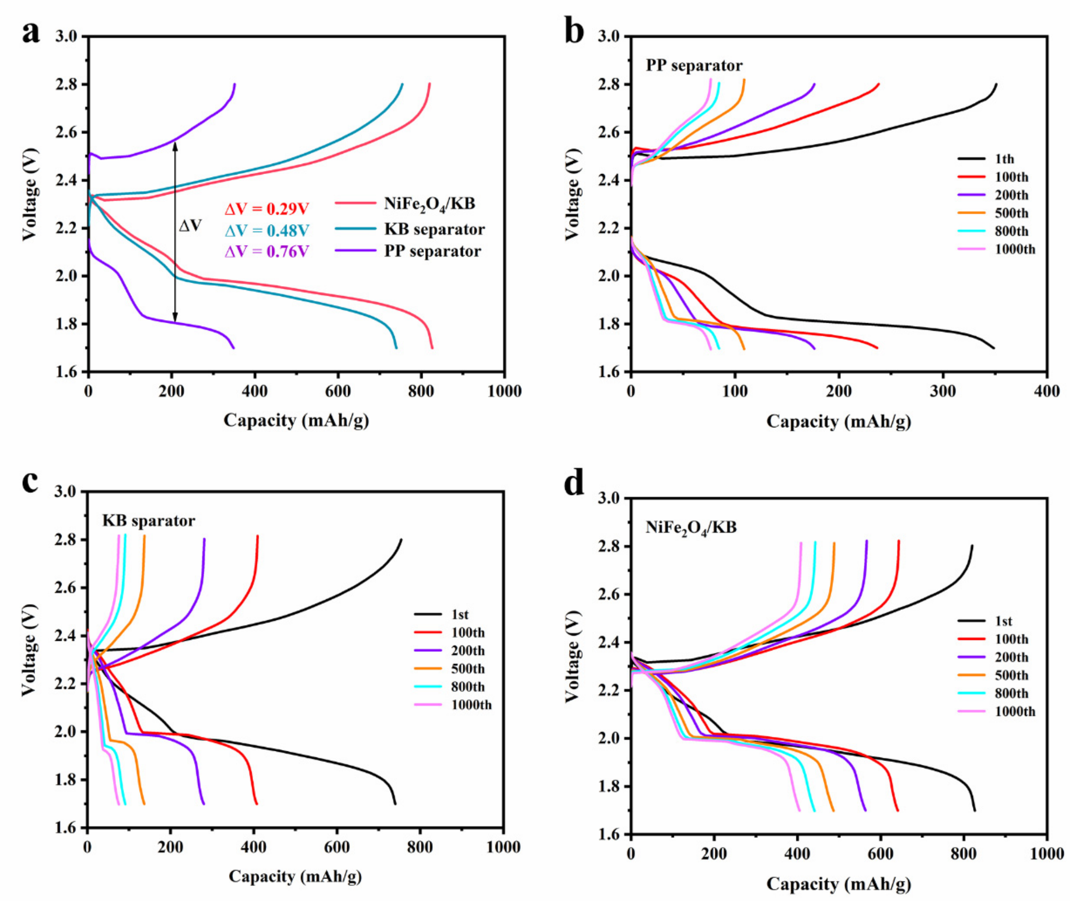

Figure S7 (a) Charge/discharge voltage profiles with different separator at 1C for the first cycle; Charge/discharge curves of cells with PP (b), KB (c) and $NiFe_2O_4$/KB separator(d) at 1C for different cycle.

S12 Summary of electrochemical performance of different modified separators for Li-sulfur batteries

Table S2. Summary of electrochemical performance of Li-S batteries configured with different modified separators and interlayers

Interlayer	Thickness (μm)	Sulfur loading (mg cm^{-2})	Sulfur content in the cathode	Rate	Cycles	Remaining Capability (mAh/g)	Decay (per cycle)	Reference
N P doped graphene	~	1.0-1.1	70%	1C	500	638	0.090%	[1]
MWCNTs/$MgAl_2O_4$	66	2.6	~	0.1C	50	~	0.759%	[2]
TiO_2/CNT	12	1.7	40%	1C	700	525	0.056%	[3]
CuS/Graphene	19.5	1.85	53%	1C	200	639	0.190%	[4]
TiO_2/KB	10.5	0.7	70%	0.5C	100	804	0.190%	[5]
KB/Ir-30	20	0.8	75%	1C	500	452	0.011%	[6]

Al_2O_3/PEI	45	1.4-1.5	80%	0.2C	200	724.9	0.224%	7
Fe_3O_4/graphene	15	0.6-0.9	60%	1C	2000	356	0.020%	8
CP/CNF	~	1.2	60%	0.3C	200	710	0.110%	9
TiO_2/Co_3O_4/NPCS	~	1.5	60%	1C	300	684.5	0.033%	10
MnO_2/CNF	2	2	80%	0.5C	200	856	0.191%	11
LaLiO2	~	1.1-1.5	70%	1C	250	872	0.052%	12
WS2/C	15	1.5	70%	1C	700	416	0.077%	13
CNF/Cos/KB	10	1.8-2.0	70%	1C	650	538	0.076%	14
MoS_2/rGO	8	1.8-2.0	70%	1C	500	368	0.116%	15
ACNF20	21	2.1-2.3	70%	0.5C	500	594	0.170%	16
$MnFe_2O_4$/AB	14.6	0.7-1	70%	1C	500	652	0.074%	17
ZnO/PPy	12.4	1.3	80%	0.2C	100	579	0.517%	18
$Li_4Ti_5O_{12}$/CNF	~	1	60%	1C	400	618	0.125%	19
Al_2O_3	14	1.6	~	0.2C	50	539.4	~	20
ZnO/graphene	71.2	1.1-1.5	80%	1C	500	641.6	0.093%	21
$NiFe_2O_4$/KB	40	0.8-1.0	70%	1C	1000	406	0.051%	This work

S13 Lithium polysulfide adsorption experiment

Polysulfide sample preparation: monomer S and Li_2S were added to an equal volume of DOL/DME solvent at a molar ratio of 5:1 and stirred at 60°C for 24h to obtain Li_2S_6 solution. Then 30 mg of KB and $NiFe_2O_4$ were added to the vials containing Li_2S_6 solution, respectively, for adsorption tests. All procedures were performed in a glove box filled with Ar gas. The adsorption capacity of the samples was examined using UV-Vis absorption spectroscopy (PE lambda 750).

S14 DFT calculation

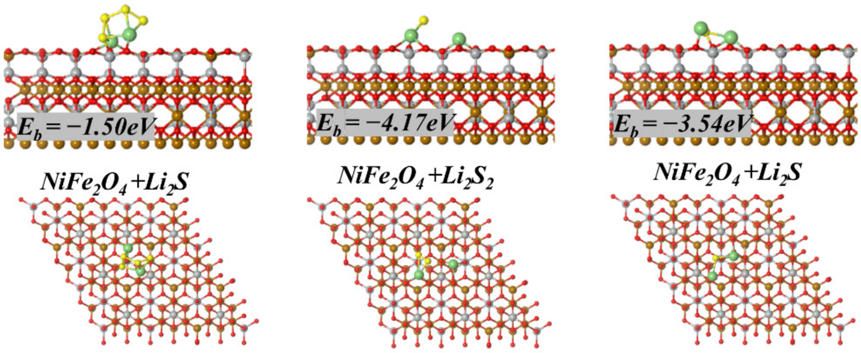

Figure S8 (a) side view of and top view of the adsorption configuration of Li_2S_x(x=1,2,4) on the $NiFe_2O_4$ (1 0 0) surface.

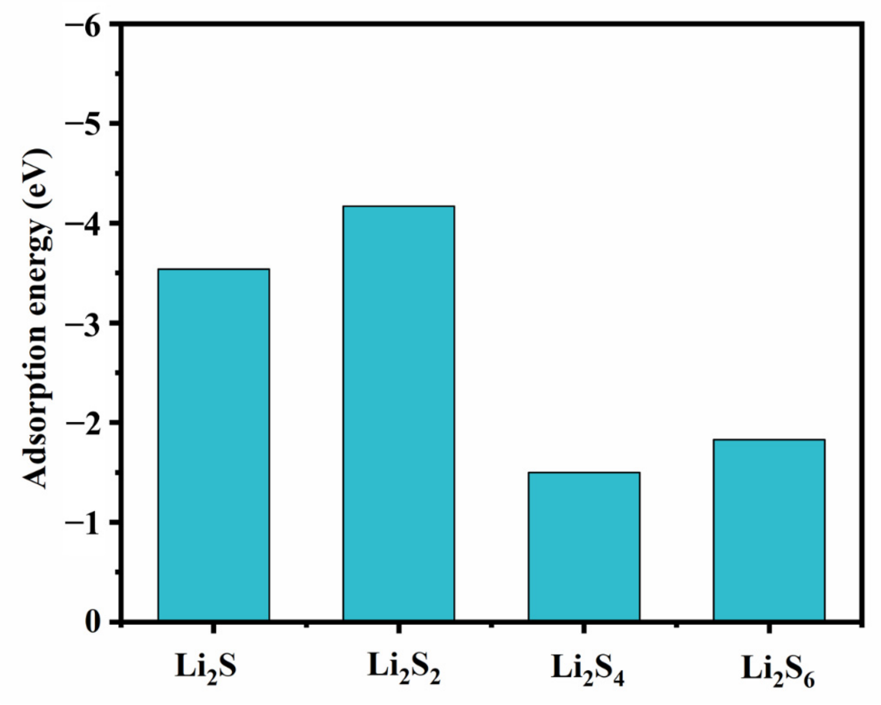

Figure S8 (b) the adsorption energy of Li_2S_x(x=1,2,4,6) on the $NiFe_2O_4$ (1 0 0) surface.

Reference

1. Gu, X.; Tong, C.-j.; Lai, C.; Qiu, J.; Huang, X.; Yang, W.; Wen, B.; Liu, L.-m.; Hou, Y.; Zhang, S., A porous nitrogen and phosphorous dual doped graphene blocking layer for high performance Li–S batteries. *Journal of Materials Chemistry A* **2015,** *3* (32), 16670-16678.
2. Raja, M.; Suriyakumar, S.; Angulakshmi, N.; Manuel Stephan, A., High performance multi-functional trilayer membranes as permselective separators for lithium–sulfur batteries. *Inorganic Chemistry Frontiers* **2017,** *4* (6), 1013-1021.
3. Yang, L.; Li, G.; Jiang, X.; Zhang, T.; Lin, H.; Lee, J. Y., Balancing the chemisorption and charge transport properties of the interlayer in lithium–sulfur batteries. *Journal of Materials Chemistry A* **2017,** *5* (24), 12506-12512.
4. Li, H.; Sun, L.; Zhao, Y.; Tan, T.; Zhang, Y., A novel CuS/graphene-coated separator for suppressing the shuttle effect of lithium/sulfur batteries. *APPLIED SURFACE SCIENCE* **2019,** *466*, 309-319.
5. Shan, L.; Yurong, C.; Jing, Y.; Feixia, R.; Jun, W.; Babu, S.; Xin, Y.; Junkuo, G.; Juming, Y., Entrapment of polysulfides by a Ketjen Black & mesoporous TiO2 modified glass fiber separator for high performance lithium-sulfur batteries. *JOURNAL OF ALLOYS AND COMPOUNDS* **2019,** *779*, 412-419.
6. Zuo, P.; Hua, J.; He, M.; Zhang, H.; Qian, Z.; Ma, Y.; Du, C.; Cheng, X.; Gao, Y.; Yin, G., Facilitating the redox reaction of polysulfides by an electrocatalytic layer-modified separator for lithium–sulfur batteries. *Journal of Materials Chemistry A* **2017,** *5* (22), 10936-10945.
7. Zhu, F.; Liu, J.; Zhao, H.; Li, J.; Li, Q.; Xi, Y.; Liu, M.; Wang, C., Preparation and Performance of Porous Polyetherimide/Al2O3 Separator for Enhanced Lithium-Sulfur Batteries. *ChemElectroChem* **2019,** *6* (11), 2883-2890.
8. Liu, Y.; Qin, X.; Zhang, S.; Liang, G.; Kang, F.; Chen, G.; Li, B., Fe3O4-Decorated Porous Graphene Interlayer for High-Performance Lithium-Sulfur Batteries. *ACS Appl Mater Interfaces* **2018,** *10* (31), 26264-26273.
9. Li, Q.; Liu, M.; Qin, X.; Wu, J.; Han, W.; Liang, G.; Zhou, D.; He, Y.-B.; Li, B.; Kang, F., Cyclized-polyacrylonitrile modified carbon nanofiber interlayers enabling strong trapping of polysulfides in lithium–sulfur batteries. *Journal of Materials Chemistry A* **2016,** *4* (33), 12973-12980.
10. Fan, C.-Y.; Liu, S.-Y.; Li, H.-H.; Shi, Y.-H.; Wang, H.-C.; Wang, H.-F.; Sun, H.-Z.; Wu, X.-L.; Zhang, J.-P., Synergistic mediation of sulfur conversion in lithium–sulfur batteries by a Gerber tree-like interlayer with multiple components. *Journal of Materials Chemistry A* **2017,** *5* (22), 11255-11262.
11. Lai, Y.; Wang, P.; Qin, F.; Xu, M.; Li, J.; Zhang, K.; Zhang, Z., A carbon nanofiber@mesoporous δ-MnO2 nanosheet-coated separator for high-performance lithium-sulfur batteries. *Energy Storage Materials* **2017,** *9*, 179-187.
12. Bizuneh, G. G.; Fan, J.; Sun, C.; Xiangfei, Y.; Xue, F.; Deng, D.; Lei, J.; Lin, X.; Jia, Y.; Yang, J.; Yan, H.; Wang, X.; Zheng, M.; Dong, Q., LaLiO2-Based Multi-Functional Interlayer for Enhanced Performance of Li-S Batteries. *JOURNAL OF THE ELECTROCHEMICAL SOCIETY* **2019,** *166* (2), A68-A73.
13. Ali, S.; Waqas, M.; Jing, X.; Chen, N.; Chen, D.; Xiong, J.; He, W., Carbon-Tungsten Disulfide Composite Bilayer Separator for High-Performance Lithium-Sulfur Batteries. *ACS Appl Mater Interfaces* **2018,** *10* (46), 39417-39421.
14. Yang, Y.; Wang, S.; Zhang, L.; Deng, Y.; Xu, H.; Qin, X.; Chen, G., CoS-interposed and Ketjen black-embedded carbon nanofiber framework as a separator modulation for high performance Li-S batteries. *CHEMICAL ENGINEERING JOURNAL* **2019,** *369*, 77-86.
15. Tan, L.; Li, X.; Wang, Z.; Guo, H.; Wang, J., Lightweight Reduced Graphene Oxide@MoS2 Interlayer as Polysulfide Barrier for High-Performance Lithium-Sulfur Batteries. *ACS Appl Mater Interfaces* **2018,** *10* (4), 3707-3713.
16. Chung, S.-H.; Han, P.; Singhal, R.; Kalra, V.; Manthiram, A., Electrochemically Stable Rechargeable Lithium-Sulfur Batteries with a Microporous Carbon Nanofiber Filter for Polysulfide. *Advanced Energy Materials* **2015,** *5* (18).
17. Wang, Y.; Guo, X.; Chen, C.; Wang, Y.; Li, Q.; Wu, Z.; Zhong, B.; Chen, Y, Alleviating the shuttle effect via bifunctional MnFe2O4/AB modified separator for high performance lithium sulfur battery. *ELECTROCHIMICA ACTA* **2020,** *354*.
18. Yin, F.; Ren, J.; Zhang, Y.; Tan, T.; Chen, Z., A PPy/ZnO functional interlayer to enhance electrochemical performance of lithium/sulfur batteries. *Nanoscale Res Lett* **2018,** *13* (1), 307.

19. An, D.; Shen, L.; Lei, D.; Wang, L.; Ye, H.; Li, B.; Kang, F.; He, Y.-B., An ultrathin and continuous $Li_4Ti_5O_{12}$ coated carbon nanofiber interlayer for high rate lithium sulfur battery. *Journal of Energy Chemistry* **2019,** *31*, 19-26.
20. Zhang, Z.; Lai, Y.; Zhang, Z.; Zhang, K.; Li, J., Al_2O_3-coated porous separator for enhanced electrochemical performance of lithium sulfur batteries. *ELECTROCHIMICA ACTA* **2014,** *129*, 55-61.
21. Shi, N.; Xi, B.; Feng, Z.; Wu, F.; Wei, D.; Liu, J.; Xiong, S., Insight into different-microstructured ZnO/graphene-functionalized separators affecting the performance of lithium–sulfur batteries. *Journal of Materials Chemistry A* **2019,** *7* (8), 4009-4018.
